# Formation of trihalomethanes as disinfection byproducts in herbal spa pools

**DOI:** 10.1038/s41598-018-23975-2

**Published:** 2018-04-09

**Authors:** Hoda Fakour, Shang-Lien Lo

**Affiliations:** Graduate Institute of Environmental Engineering National Taiwan University No. 1, Sec. 4, Roosevelt Rd., Taipei, 10617 Taiwan (R.O.C.)

## Abstract

Herbal spa treatments are favorite recreational activities throughout the world. The water in spas is often disinfected to control pathogenic microorganisms and guarantee hygiene. However, chlorinated water may cause the formation of disinfection byproducts (DBPs). Although there have been many studies on DBP formation in swimming pools, the role of organic matter derived from herbal medicines applied in herbal spa water has been largely neglected. Accordingly, the present study investigated the effect of herbal medicines on the formation of trihalomethanes (THMs) in simulated herbal spa water. Water samples were collected from a spa pool, and then, disinfection and herbal addition experiments were performed in a laboratory. The results showed that the organic molecules introduced by the herbal medicines are significant precursors to the formation of THMs in spa pool water. Since at least 50% of THMs were produced within the first six hours of the reaction time, the presence of herbal medicines in spa water could present a parallel route for THM exposure. Therefore, despite the undeniable benefits of herbal spas, the effect of applied herbs on DBP formation in chlorinated water should be considered to improve the water quality and health benefits of spa facilities.

## Introduction

Since 1968, when Roy Jacuzzi created the world’s first integrated whirlpool bath, hot tubs and spas have transformed bathing into an excellent relaxation technique. A spa pool consists of a relatively small volume of warm water (35 °C – 39 °C) in which bathers sit instead of swim. Currently, there are many treatments for different purposes to enhance the quality of life, with improved health and less stress. Health clubs combining herbal spas, water parks, and swimming pools are big businesses throughout the world^1^.

“Spa Therapy” presently indicates a wide range of treatments that have become very popular as a therapy for pain and injuries during a recovery period. There are several different traditional spa treatment methods, including massages, facials, body wraps, and exfoliation. Although useful, these conventional therapies have drawbacks that have led to alternative therapies through herbal medicine. According to the World Health Organization (WHO), approximately 25% of medicines are obtained from plants that were first used in traditional systems of medicine^2^.

To improve the remedial and health benefits of spa water, herbs and fruits such as ginseng, rosemary, rose, lavender, ginger, green tea, mint, and shaddock are commonly added to the water^3^. It is believed that soaking the body in a basin of soothing herbal infusion is a powerful remedy for different pain relief purposes. Bathing in herbal-scented water can help reduce stress, protect the skin and relieve muscles at the end of busy days. Since herbal baths are widely used to treat skin conditions, including acne, dermatitis, eczema, scalp itching, flaking and dandruff, a spa pool must be managed carefully to ensure that the water quality does not deteriorate. Therefore, pool water disinfection is a critical precautionary measure to prevent microbial pathogens^4^.

Among different sterilization approaches, one of the most economical methods is chlorination, which is commonly used in pool water. However, chlorination may lead to disinfection by-product (DBP) formation, including trihalomethanes (THMs) and haloacetic acids (HAAs), which are the commonly regulated carcinogenic DBPs in drinking water^5^. Epidemiological pieces of evidence have linked the exposure to DBPs in chlorinated water to the bladder and colorectal cancer^6,7^.

The formation of DBPs in swimming pools depends on several factors, including the sum of precursors in the water (such as total organic carbon (TOC)), the number of people in the pool, the water temperature, and the chlorine dose^8^.

Since the chemistry of swimming pool water is complex, the exposure pathways and potential health risks are still relatively unknown and have considerable uncertainties.

Among the different DBPs, particular concerns are associated with the THMs because they have been recognized as potentially hazardous and are the major by-products of chlorination^9^. THMs are also common DBPs in pools^10,11^. Chloroform is widely found in municipal water supplies that are disinfected by chlorination^12^. THMs are typically more dominant than HAAs, and in general, DBPs that react with chlorine are more prominent than other types^13^. It has been shown that the consumption of water containing high concentrations of THMs may lead to liver, kidney, and central nervous system problems^14^. Besides, THMs are relatively persistent in water supplies and distribution systems. The THMs half-life range from 1 to 65 days with chloroform being the most persistent species^15,16^. The WHO recommended a maximum concentration of 100 µg/L of total THMs (TTHM) for all types of pools^17^. In some countries such as Taiwan, each city establishes its own water quality standards for spas. The general requirement for the concentration of free residual chlorine (FRC) in pool water ranges between 1.0 and 3.0 mg/L. Most spas are often chlorinated with bleach powder, bleach liquid, or sodium hypochlorite to comply with these policies and guidelines^1^.

Several studies have estimated THM exposure in pool environments. Some publications reported that high concentrations of several DBPs are present in the air and water of pools that utilize chlorination as a disinfection process. Beech *et al*.^18^ reported an average concentration of 125 µg/L for the TTHM (mainly chloroform) in the water of 101 pools in Miami, Florida (United States of America (USA)), and Sandel^19^ reported an average chloroform level of 67 µg/L in 114 tested residential pools in the USA. Fantuzzi *et al*.^20^ examined five indoor swimming pools in Italy and reported an average THM concentration of 40 µg/L. In the study of Aggazzotti and Predier^21^, high levels of volatile halogenated organics (mainly THMs) were observed in swimming pools as a consequence of chlorination with sodium hypochlorite. They also showed that the DBP levels in pool water are higher than in drinking water. Weaver *et al*.^22^ showed that approximately 45% of the TTHM measurements from 11 indoor chlorinated swimming pools exceeded the EPA maximum contaminant levels (MCL) of 80 µg/L. More importantly, it has been estimated that approximately 80% of THM uptake for swimmers is through dermal absorption^23^. In a study by Chen *et al*.^24^, the concentration of chloroform in the swimming pools of southern Taiwan was reported to be 9.81 µg/L.

There is a wide variation for different studies in the reported levels of DBPs in swimming pools, but overall, these studies indicate that DBP exposure in pool water can be more substantial than in drinking water^17,21,25^.

Although many studies have been conducted to evaluate the presence of DBPs in swimming pools, very little attention has been paid to investigate the generation of chlorinated by-products in herbal spa water, which is one of the most common recreational therapies in spa facilities. Bath bags and herbal medicines are becoming increasingly popular around the world. Herbal infusions are usually prepared by soaking dried plant materials in hot water, resulting in the extraction of different essential oils and organic compounds. Despite the undeniable benefits of herbal spas, the effect of organic matter derived from herbal medicine on THM formation in chlorinated water has been largely neglected. The presence of herb-derived organic matter in the chlorinated water of spa pools and bath centers may potentially enhance the formation of carcinogenic DBPs. To the best of our knowledge, this is the first study that investigates the role of herbal medicines in THM formation, and this information can explain the additional exposure pathways of THMs in water-related activities.

Thus, the purpose of this study is to provide a better understanding of the effect of herbal spa infusions and tea bath bags on the occurrence and formation of THMs in simulated herbal spa water. Different commonly used herbal medicines, including ginger (*Zingiber officinale*), Asian ginseng (*Panax ginseng*), mint (*Mentha spicata*), and rosemary (*Rosmarinus officinalis*), were applied to simulate real herbal spa conditions. These herbs are among the most common herbal medicines used for different purposes in spa facilities. The oily resin from the roots of ginger contains many bioactive components that are believed to exert a variety of significant pharmacological and physiological activities. A ginger bath is widely used as a supplementary healing therapy for different health problems, such as skin diseases, joint pain, hypertension, insomnia or diarrhea^26,27^. In ancient times, ginseng was used as an aphrodisiac, an anti-aging agent, and an energizer. Currently, ginseng is also used to enhance energy levels and is used as an antioxidant^28,29^. Ancient physicians have used mint and rosemary for medical purposes. Mint has been used as a digestive aid and as a treatment for colds, cough, fever, and diarrhea^30^. A rosemary bath is believed to benefit mental and physical health; with anti-spasmodic and rubefacient properties, this aromatic plant is one of the most widely used herbal medicines for muscle pain^31^.

In the current study, various chlorination levels along with different herbal medicines were applied to real spa water to simulate herbal spa conditions. Exploring the role of widely used herbal medicines on the THM concentrations in chlorinated spas and hot tubs would be beneficial for developing simple prevention strategies and for explaining the mechanism of THM formation in herbal spas. Ultimately, effective countermeasures can be further developed by understanding these interrelationships.

## Results and Discussion

### Water quality analysis

The spa water investigated in the present study was obtained from an indoor spa with an irregular shaped pool lined with glazed tiles. The water samples taken from the spa pool were disinfected with different NaOCl concentrations in the laboratory. Table 1 summarizes the measured values (mean ± SD) of the temperature, pH level, conductivity, FRC, dissolved oxygen content (DOC), UV, and specific UV absorbance (SUVA) of the water samples together with the TTHM concentration. The bromide and iodide ion concentrations of the collected samples were 0.28 and 0.008 mg/L, respectively. The THM levels were obtained in the laboratory after mixing for 30 minutes to achieve the different disinfectant levels. To ensure the reliability of the analysis results, each sample was tested in triplicate.

**Table 1:** Water quality parameters of the spa water samples.

Disinfectant level (mg L^−1^)	Water quality parameters								
	pH	Temperature (°C)	Conductivity (mS cm^−1^)	DOC (mg/L)	UV_254_ (1/cm)	SUVA (mg^−1^·m^−1^)	FRC (mg L^−1^ Cl_2_)	Turbidity (NTU)	TTHM (µ/L)
0	7.1 ± 0.18	39 ± 0.31	0.58 ± 0.001	2.9 ± 0.21	0.050 ± 0.003	1.72 ± 0.11	0.00 ± 0.00	≤1.0	NA
0.5	7.2 ± 0.22	38 ± 0.24	0.62 ± 0.002	2.7 ± 0.17	0.041 ± 0.002	1.51 ± 0.07	0.24 ± 0.03	≤1.0	45 ± 3.21
1.5	7.0 ± 0.31	39 ± 0.11	0.55 ± 0.001	1.8 ± 0.26	0.023 ± 0.003	1.27 ± 0.09	0.91 ± 0.10	≤1.0	59 ± 4.34
3.0	7.2 ± 0.24	38 ± 0.27	0.70 ± 0.002	0.8 ± 0.14	0.008 ± 0.001	1.0 ± 0.08	2.2 ± 0.21	≤1.0	72 ± 5.72

^*^Not applicable.

The disinfectant concentration had no significant effect on pH, temperature, conductivity, and turbidity (P-value > 0.10). Increasing the concentration of the disinfectant caused a decreased DOC level and an increased FRC. The SUVA value also decreased with an increasing disinfectant level. The reduction in the SUVA value is attributed to the removal of humic substances according to the chlorine dosage. An increasing chlorine dosage damages the aromatic structure of the organic matter and results in the reduction of the SUVA values^32^. When the chlorine dosage increased from 0.5 mg/L to 3 mg/L, the SUVA value of the spa water decreased by up to 34%. This trend is similar to that observed from the UV_254_ data in this experimental study.

Although TTHM was detected after the addition of disinfectant, the average THM concentration did not exceed the regulated MCL for TTHM established by the WHO of 100 µg/L^17^ for spa pools.

**Figure 1:**
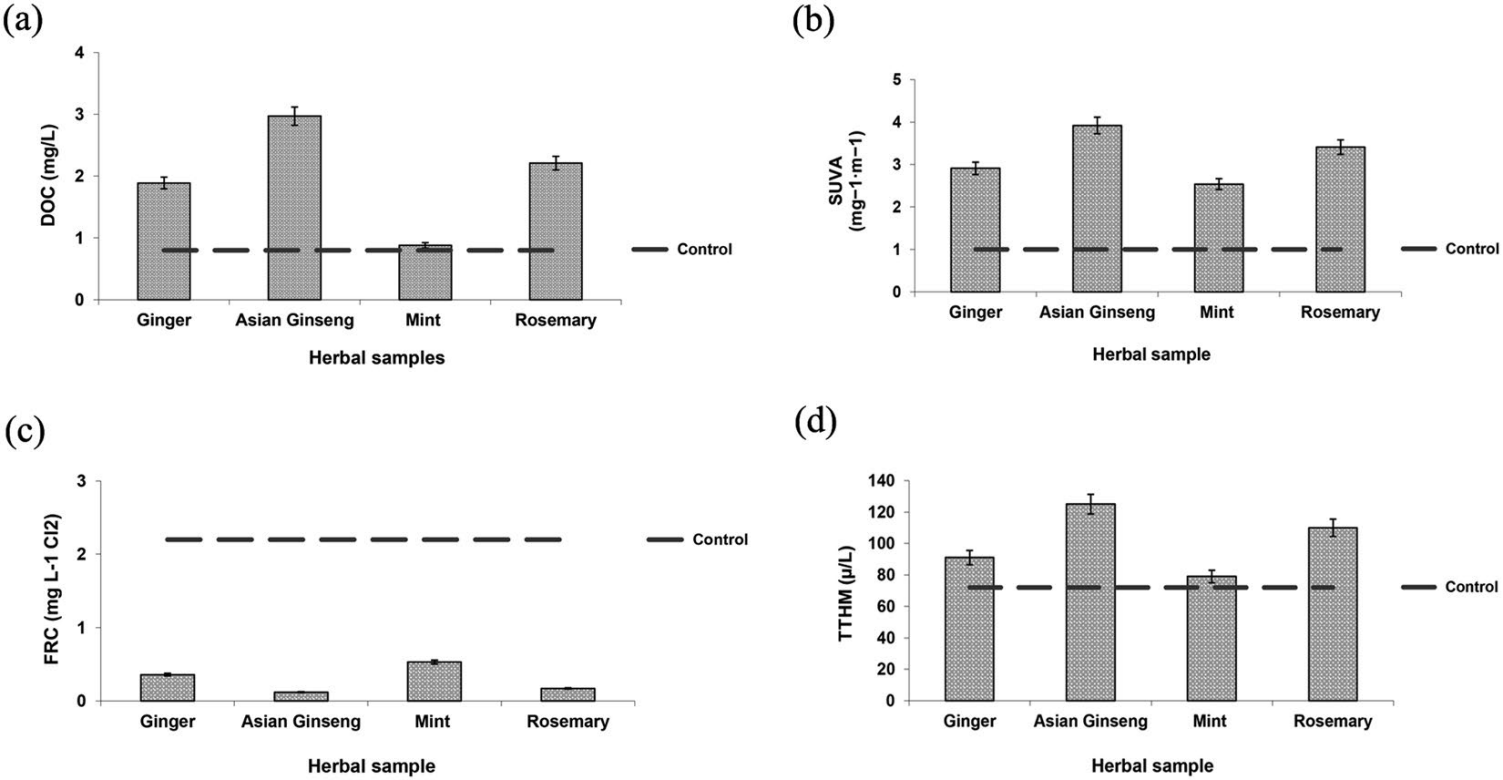
THM reactivity of the different samples after the addition of herbal species (mass concentration of herbal medicines: 10%; chlorine dose: 3 mg/L; reaction time 72 hours).

Among the different water quality parameters, DOC, SUVA_254_, FRC, and TTHM were chosen to represent the THM reactivity of the different samples after the addition of species of herbs with a 10% mass concentration of herbal medicines at a 3 mg/L chlorine dose after 72 hours of reaction (Fig. 1). Control samples without the addition of herbal medicines but with chlorination (3 mg/L) were also prepared at the same time for comparison.

The difference between TTHM formation in the herb-containing sample and the control sample (with no herbal addition) was significant in all cases (P-value < 0.001), except for the mint-containing sample (P-value = 0.12). The spa samples that contained Asian ginseng and rosemary showed the highest TTHM levels, which exceeded the maximum concentration established by the WHO for all types of pools (100 µg/L)^17^. Since the guidelines for drinking water quality can also be used to screen the potential risks arising from swimming pools, and similar environments, most studies regarding the DBP concentrations in swimming pools consider the regulations established for tap water. The MCLs of the total THMs in tap water should not be higher than 80 μg/L (USEPA phase I) with the MCL goal (MCLG) for the total THM concentration at 40 μg/L (USEPA phase II)^33,34^. The mean concentration of TTHM in all herbal spa samples was higher than the mean THM levels in swimming pool water in several other studies^35–37^. The potential to generate high levels of chlorinated by-products on a daily basis in the herbal spa community could represent an environmental and health assessment problem because long-term exposure to high THM levels (>50 µg/L) could result in serious health problems, such as bladder cancer^38^.

SUVA is also an excellent predictive parameter for the aromatic carbon content of natural organic matter (NOM) in water and, consequently, the THM formation potential^39^. SUVA values of less than two indicate a higher content of hydrophilic fractions with low UV absorbance, chlorine demand and THM formation potential (THMFP). Values between two and four show a mixture of hydrophobic and hydrophilic compounds with average UV absorbance, higher chlorine demand, and THMFP. SUVA values higher than four are indicative of a highly aromatic hydrophobic fraction associated with high UV absorbance, chlorine demand, and THMFP^40^. The hydrophobic matter is rich in aromatic compounds and is the primary precursor of DBPs^41^. Korshin *et al*.^42^ indicated that with increasing SUVA values, the formation potential of THMs also increases.

All herbal medicines contain both hydrophobic and hydrophilic compounds, and samples containing Asian ginseng showed the highest SUVA value. More than ten phenolic compounds, including caffeic acid, ferulic acid, vanillic acid, p-hydroxybenzoic acid, gentisic acid, and syringic acid, have been reported in ginseng plants^43–45^. Despite the various biological properties, such as antioxidant and anticancer effects^46^, of the phenolic compounds present in ginseng, the phenolic/aromatic fractions of NOM are believed to be reactive sites for by-product formation^47^ and one of the major precursors to the formation of THMs^48^. Furthermore, the temperature of spa water is usually higher than that of swimming water, which facilitates the generation of more volatile organic components from the ginseng samples^49^.

### Effect of Chlorination dosage on the DOC concentration

**Figure 2:**
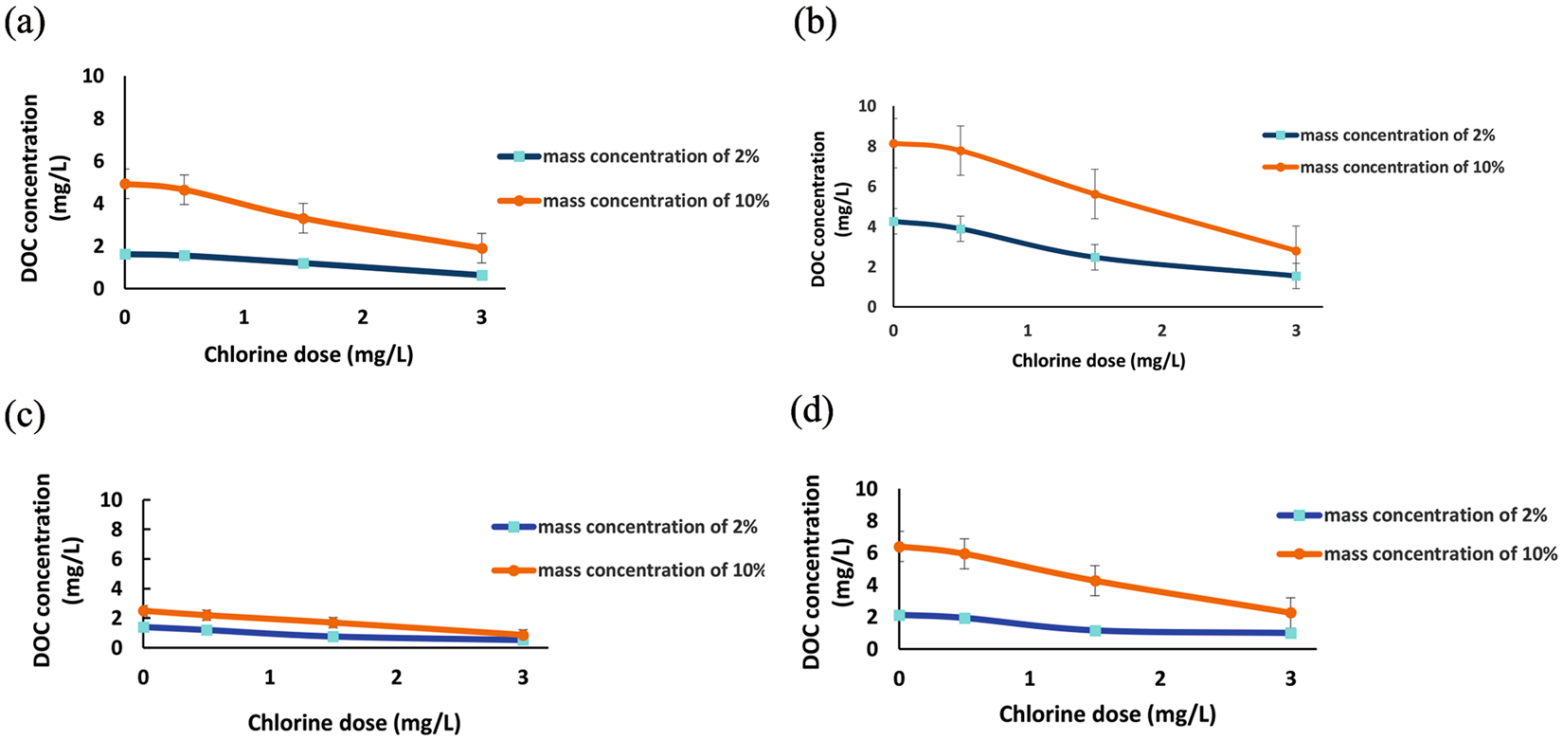
Effect of the chlorination dosage on the DOC concentration of (**a**) ginger, (**b**) Asian ginseng, (**c**) mint, and (**d**) rosemary contained in the spa water at different mass concentrations of the applied herb.

Since water treatment for NOM removal would minimize THM formation by reducing the amount of NOM available to react with chlorine, it is essential to investigate the NOM reduction at different disinfectant dosages to estimate the disinfection by-product formation potential (DBPFP) under realistic conditions. The presence of herbal medicine, as the source of organic matter, at different mass concentrations consequently affects the DBPFP based on the applied disinfectant level. Figure 2 shows the effect of chlorination dosage on the DOC concentration derived from herbal medicines in spa water samples with different mass concentrations of applied herb. The results show that with an increase in the NaOCl dosage from 0 to 3 mg/L, the DOC concentration decreased approximately 50–63% and 59–65% for 2 and 10% mass concentrations, respectively. The water that contained the Asian ginseng showed the highest DOC concentration at each disinfectant dosage. It has been demonstrated that the significant constituents of ginseng are carbohydrates, accounting for over 50% (w/w dry basis) and including starch, cellulose, glycosides^50^, and approximately 11% protein^51^. In each mass concentration, DOC was almost unchanged with low levels of NaOCl, suggesting that the aromatic ring was not well mineralized by NaOCl. However, when DOC decreased with increasing NaOCl, the aromatic ring was broken up, and the fraction of organic carbon was mineralized by NaOCl^52^. In commercial applications, low or moderate oxidation conditions are applied. Under these circumstances, the NOM is partially oxidized, and high-molecular-weight compounds are transformed into smaller and more biodegradable compounds, such as aldehydes and carboxylic acids^53,54^. These changes in the chemical characteristics of the NOM also result in a reduction in the organic matter concentrations and an alteration in the characteristics of the DBP precursor materials and potentially their reactivity with chlorine^55^.

### Role of the chlorine demand

**Figure 3:**
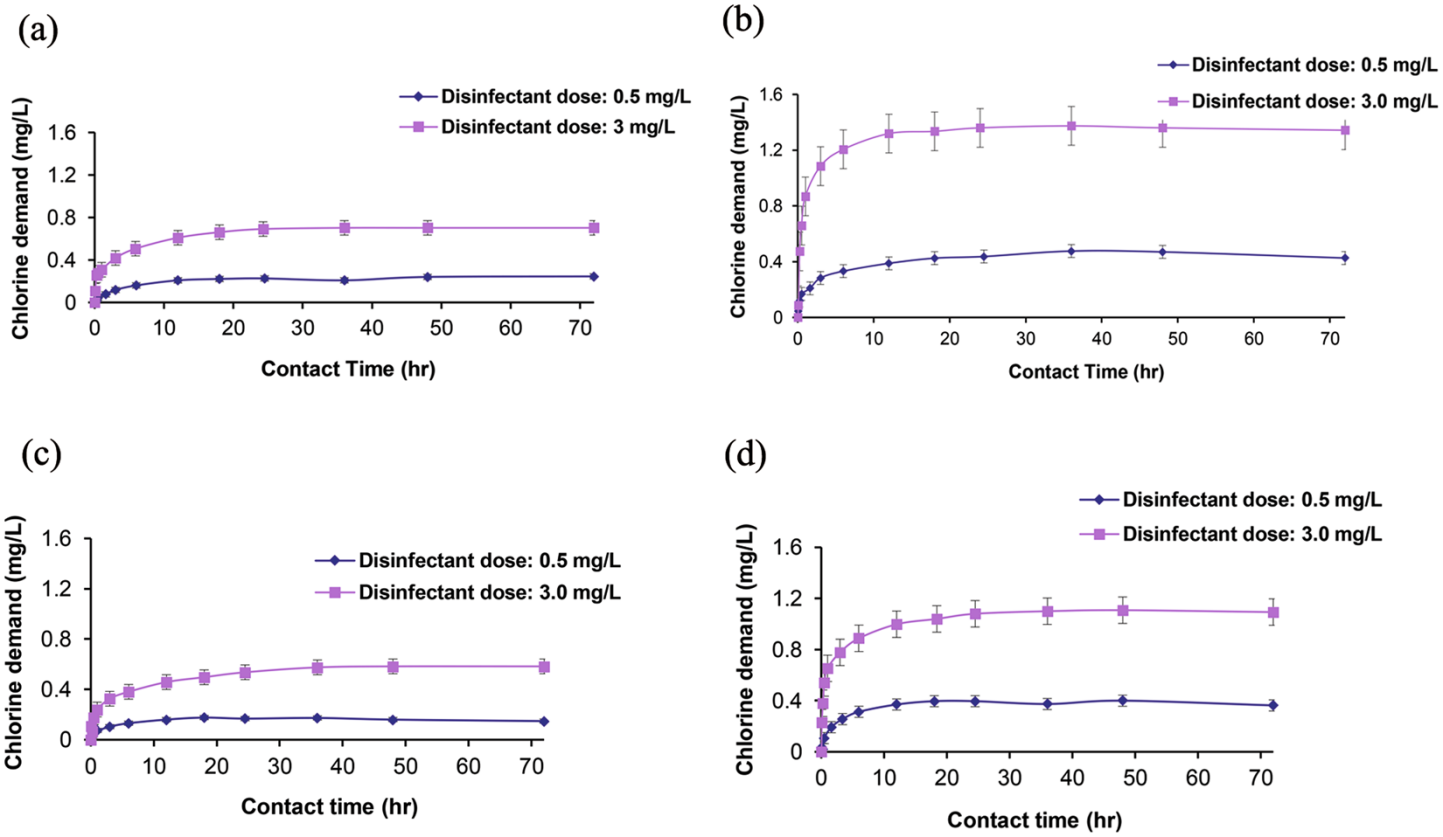
Chlorine demand of (**a**) ginger, (**b**) Asian Ginseng, (**c**) mint, and (**d**) rosemary contained in the spa water at various disinfectant dosages.

The changes in the chlorine demand could potentially enhance the formation of DBPs. The measured chlorine demand profile of a representative set of the tested water samples is illustrated in Fig. 3. For all sets of initial conditions, the chlorine demand increases with increasing contact time and approaches a constant value, which is the final chlorine demand. This result is not surprising because more contact time causes a commensurate increase in demand. Then, the increase in the contact time beyond the “ultimate” demand does not lead to increased chlorine loss. It also appears that the initial slope is steepest for the sample containing ginseng, with the shortest contact time required to reach the final chlorine demand.

Figure 3 also shows that the rate of chlorine demand is most rapid during the first 24 hours and reached its ultimate demand at a maximum of 36 hours. Although different plants have unique chlorine demands, even with similar organic carbon contents and concentrations, the higher percentage of active organic compounds (such as amino acids) in Asian ginseng^56^ might explain the higher chlorine demand and the more rapid reaction with free chlorine. A more interesting result is that higher initial chlorine concentrations resulted in higher chlorine demands. In each of the chlorine demand curves in Fig. 3, the initial chlorine concentration of 3 mg/L produced a greater final chlorine demand than did lower initial chlorine concentrations for each sample. Lin and Evans^57^ also reported that the higher chlorine doses of sewage effluents produced greater chlorine demands at all contact times. This result could be because the solutions with a higher oxidation potential (higher free chlorine concentration) either reacted longer with the organic material or continued to react with intermediates formed by the initial oxidation reaction^58^.

### Effect of chlorine dosage and organic mass concentration on the TTHM levels

Table 2 shows the impact of the chlorination dosage and mass concentration of the applied herbs on the TTHM levels in different herbal spa samples. As the chlorine content and organic mass concentration of the herbal medicines increased, the THM concentration also increased. Note that herbal spa pools contain organic matter not only from used herbal medicines but also from the users’ bodies, such as sweat, urine and other compounds applied to the skin. These compounds include various nitrogenous organic materials such as urea, ammonia and amino acids that may potentially react with chlorinated water in the pool to form new DBPs^59^. More importantly, the temperature of spa water is usually higher than the temperature of the water in typical swimming pools. It is known that higher temperatures accelerate the reaction rate of the chlorine and organic content in water, and there is a strong relationship between the THMFP and water temperature^60^.

**Table 2:** Effect of the chlorination dose and mass concentration of the applied herbs on the TTHM levels (µg/L) in different herbal spa samples.

Applied herb	Mass conc. (%)	NaOCl dosage (mg/L)		
		0.5	1.5	3
Ginger	2	52 ± 2.61	69 ± 3.10	85 ± 4.33
	10	59 ± 2.34	79 ± 5.23	91 ± 4.52
Ginseng	2	83 ± 3.42	98 ± 7.68	115 ± 6.27
	10	75 ± 2.51	112 ± 8.91	125 ± 7.43
Mint	2	49 ± 1.92	65 ± 5.37	76 ± 5.08
	10	51 ± 3.16	68 ± 4.29	79 ± 5.95
Rosemary	2	64 ± 4.20	97 ± 7.46	101 ± 7.86
	10	79 ± 5.32	102 ± 8.83	110 ± 8.47

### Specific THM formation potential (STHMFP)

**Figure 4:**
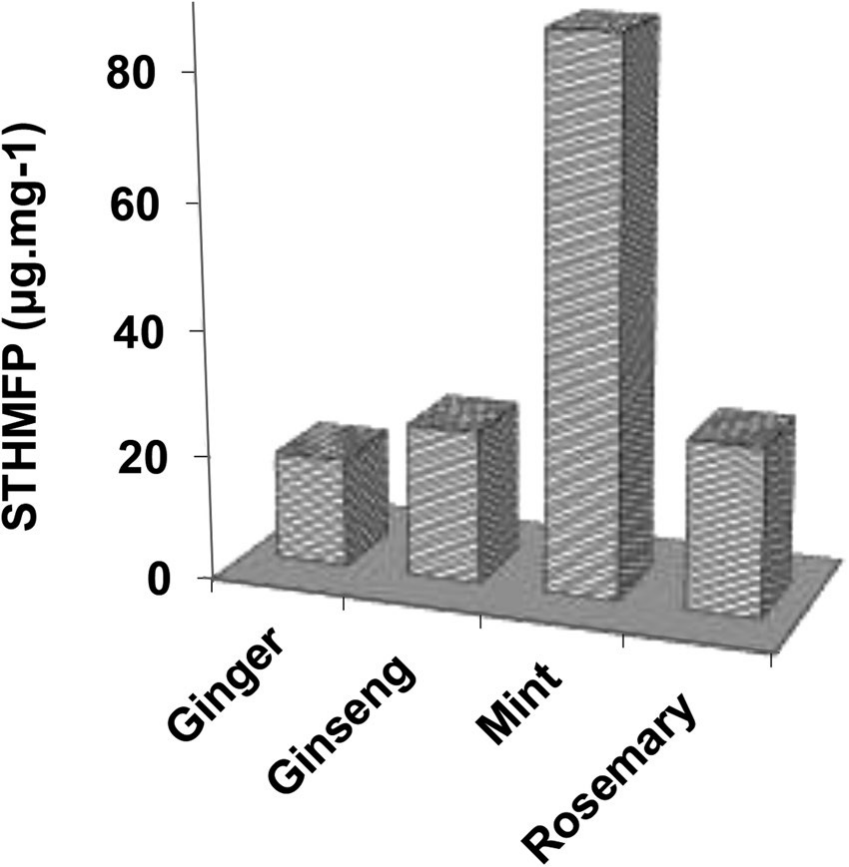
Values of the STHMFP (µg.mg^−1^) from various herbal spa water samples at a 10% mass concentration.

The THMFP is a useful parameter indicating the maximum THM content likely to be produced when chlorine reacts with organic matter present in the water sample. The THMFP is defined as the difference between the THM concentration measured 30 minutes after chlorination and the THM concentration measured after herbal addition. To measure the reactivity of the DOC to form THMs, carbon-normalized parameter, namely, the specific trihalomethane formation potential (STHMFP) (μg.mg^−1^), which is the THMFP divided by the DOC from the herbal spa samples, was calculated (Fig. 4). As seen, the mint-containing sample shows a higher STHMFP, indicating the higher reactivity of the THM precursors per mg of DOC. This result could be because although the DOC concentration of the ginseng sample was higher than those of the other herbal medicines (Fig. 1), there appear to be more reactive sites for THM formation per mg of organic carbon in the mint-containing spa water sample.

### Effect of contact time on the THM concentration

**Figure 5:**
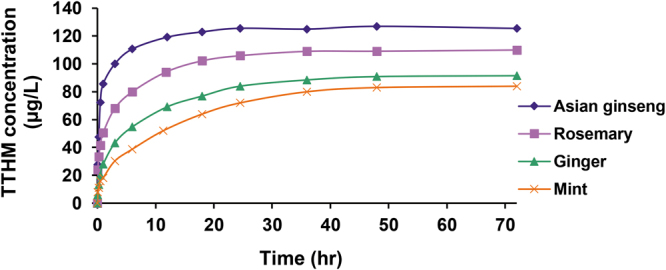
Formation of the TTHM as a function of reaction time with a chlorine dosage of 3 mg/L for different herbal spa samples.

Figure 5 shows the formation of THMs in herbal spa water samples as a function of contact time. THM formation commonly proceeded in two stages: An initial phase that was completed rapidly within the first few hours and a slower step marked by a steady rate of increase. During the slowest reaction (mint-containing sample), approximately 50% of the THMs were produced within the first 6 hours, and 95% of the THM formation corresponded to 36-hour reaction time. Asian ginseng showed the fastest reaction, with a higher THM formation as the TTHM concentration reached approximately 80% of its ultimate value after three hours of reaction. Krasner *et al*.^61^ also reported that about 70–90% of the THMs were formed within the first 24 hours.

The aromatic plant-derived organic matter may contain diverse functional groups, including highly reactive groups for THM formation (so-called “fast formers”), and other groups with lower reactivity (so-called “slow formers”)^62^. Herein, the water sample containing Asian ginseng seems to contain a higher fraction of “fast formers” to create THMs.

### DBP species distribution

**Figure 6:**
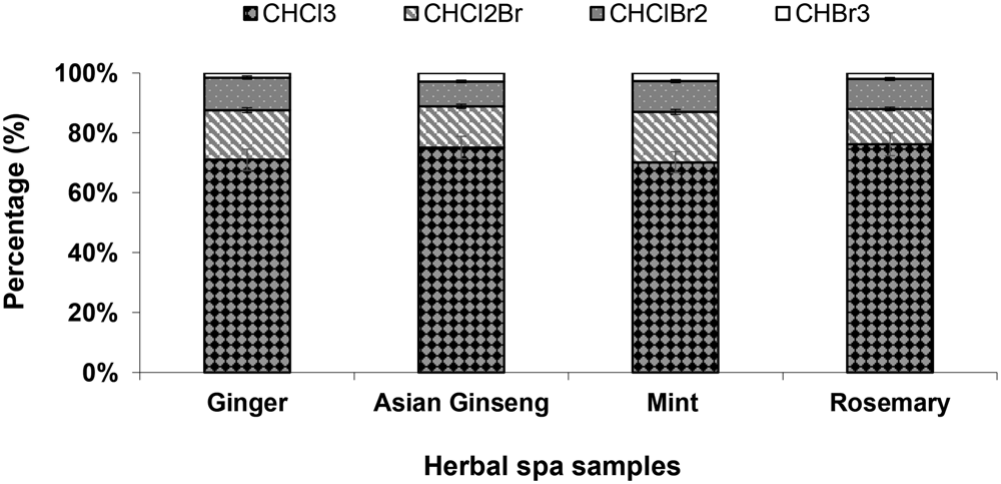
THM species distribution in different herbal spa samples.

The distribution pattern of the THM species was not significantly different in the various herbal spa samples (P-value > 0.15). Figure 6 presents the distribution pattern of the THM species in the different herbal spa samples with a 10% mass concentration after 72 hours of reaction. CHCl_3_ was found to be the dominant THM species, which accounts for an average of 73% of the total THMs in the water samples. This dominance is probably due to the formation of chloroform from the phenolic moieties, which is comparable with reactive DBP precursors found in herbs and plants^63^. A survey of 15 indoor swimming pools in Canada also revealed that chloroform was the dominant species, with concentrations ranging from 12.9 to 215 µg/L with a mean value of 55.2 µg/L^64^.

Unlike other studies concerning the THM distribution in different tea steaming and daily beverage samples^65,66^, some brominated species were also detected in the herbal spa samples in the current study. It is known that some forest soils and upland fields are considerably rich in bromine, and the conversion of forestland or upland fields into another application such as paddy or agricultural fields causes the dissolution and leaching of large amounts of bromine. This process results in the excessive absorption of bromine by the plants, leaving bromine residues that exceed the permissible limits in crops^67,68^.

Besides, Wang *et al*.^69^ revealed that in most of the Taiwan area, approximately 30%–47% of the total THMs contains bromine with an average of 10% as bromoform because of the bromide presence in the raw water sources.

Taiwan was also reported to consume approximately 4% of the world’s bromine production with a bromine concentration of 5 mg/L from ground waters due to contamination by seawater as a result of excessive pumping that exceeds the natural recharge rate^70^.

### Fourier transform infrared spectroscopy (FTIR) analysis

**Figure 7:**
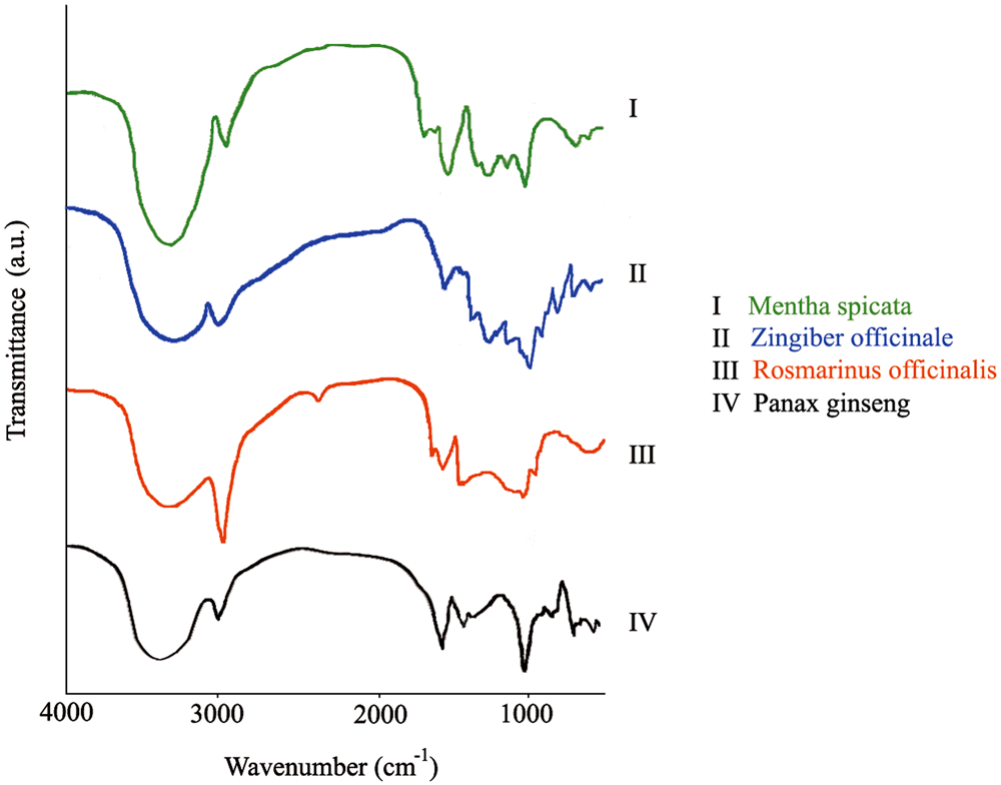
FTIR spectra of the studied herbal medicine extract.

A Fourier transform infrared (FTIR) spectrum is used to identify the active components of a material based on the peak value in the region of infrared radiation^71^. As suggested by Stevenson^72^ and Silverstein and Webster^73^, the peaks and bands that appear in FTIR spectra may be linked to the functional groups present in the studied organic materials. The FTIR spectrum of the applied herbal extract is shown in Fig. 7. The results of the functional group analysis reveal the existence of various characteristic functional groups in the plant samples. Similar absorption bands of the main functional groups in the FTIR spectra were identified in all the analyzed herbal medicines. The broad band centered between 3410 and 3371 cm^−1^ corresponds to the O-H stretching of hydroxyl groups (alcohols, phenols, and carboxylic acids) and N-H stretching in amines I, II and amides. The absorption band appearing between 2920 and 2850 cm^−1^ is due to the C-H vibration (stretch) of the aliphatic groups. The peak at 2324 cm^−1^ is due to alkyl C–H stretching, and the band at 1750–1620 cm^−1^ is assigned to the C=O vibration of bonded conjugated ketones, aldehydes, quinines, and esters. The apparent band at approximately 1600 cm^−1^ is attributed to aromatic C=C skeletal vibrations, the asymmetric stretching of the C=O of quinones and ketones, the symmetric stretching of COO‒ and the C=O stretching of the amide I band. The aromatic skeletal vibration of the lignocelluloses was absorbed in approximately 1520 cm^−1^. Another band noted between 1440 and 1410 cm^−1^ is attributed to the O-H in the plane bend of carboxylic acids, and the C-O stretch vibration of the carbonates. The vibrational absorption band at approximately 1380 cm^–1^ is assigned to the rocking motion of the methyl group.

The band at approximately 1250 cm^−1^ is linked to the C-H stretch and O-H deformation of the carboxyl groups and the N-H of amide II. Another obvious band centered at around 1050 cm^−1^ can be assigned to C-O stretching. A band at approximately 600 cm^−1^ represents the aromatic ring, and a peak at about 520 cm^−1^ could be due to the torsion and ring torsion of phenyl. The FTIR results demonstrate that the aromatic content, proteins, and phenolic hydroxyl groups are active functional groups in herbal medicines that could act as primary precursors for THM formation.

### Environmental implications

The results of this study demonstrated the importance of considering a parallel route for THM formation represented by the organic matter derived from herbal medicines in spa waters. Since at least 50% of the THMs were produced within the first six hours of reaction time in spa water containing herbal medicines, some general control measures should be adopted to reduce DBP exposure to bathers, including pre-swim showering, an efficient filtration system, and increasing the water exchanges^17^. Moreover, spa water users are usually positioned at the air-water interface where the accumulated concentrations of THMs exist just above the water surface, that is, in the air that users continually breathe. It was found that exposure to DBPs through the skin and respiratory system is significantly higher than ingestion through drinking water^6^. Improved ventilation is another important precautionary measure to reduce the volatile DBP exposure in pool air^74^.

Furthermore, it has been reported that people who frequently use swimming pools have an increased risk of bladder cancer compared to those who do not^6^, and DBPs are considered as partially responsible for the increased risk of melanoma cancer^75^. Even at a lower mass concentration of 2%, the TTHM level in both Asian ginseng- and rosemary-containing samples exceeded the maximum allowable limit of 100 µg/L set by the WHO, confirming the high potential of THM formation in herbal spa facilities due to the presence of a high level of organic materials introduced by conventional herbal bath bags. Mint-containing spa samples also showed higher STHMFP, indicating the presence of more reactive sites for THM formation per mg of organic carbon in the mint samples compared with the other herbal medicines. This finding should be carefully considered in designing the conditions (i.e., the dosage and type of applied herbs) of herbal spa pools. Further studies need to be conducted on spa facilities to establish a database for a better understanding of the different DBP concentrations, particularly at the gas-liquid interface of herbal spas, the impact of pool operation practices and environmental factors on DBP formation in herbal spa waters.

Ultimately, the key point is to maintain microbial disinfection during spa pool operation to minimize the formation of potentially harmful DBPs. The goal is to retain the positive therapeutic benefits and health effects of the herbal spas while reducing their potential adverse health risks.

## Methods

### Sampling procedure

Water samples were collected from a spa pool facility located in Taipei City, Taiwan. All of the sample gathering, handling, and data analysis procedures were performed following the relevant NIEA Guidelines set by the Taiwan Environmental Protection Administration^76^. Before spa water sampling, the sampling bottles were sanitized with a 1.6 M nitric acid solution or a 2 M hydrochloric acid solution and then were rinsed with DI water. Each sample was collected at a 30-cm depth below the water surface and a 1-m distance from the edge of the spa without the presence of air pockets in the bottles to prevent oxygen from entering into the samples.

### Analytical procedure

Methanol, carbon disulfide, acetone, and 1,2-dibromopropane with high purity (>99%) were purchased from Merck Chemical Company (Merck, Darmstadt, Germany). UV/visible spectrophotometry (Genesys™ 10 S, WI, USA) at 254 nm and TOC analysis (Aurora model 1030w /1088, USA) methods were used to measure the UV-absorbance and DOC levels in the samples, respectively, after filtration through 0.45-µm membrane filters. For the TOC analysis, standards of 0, 1, 2, 5, and 10 mg/L of total carbon in ultrapure water were prepared with potassium hydrogen phthalate (KHP) for all the dilutions. The SUVA was calculated as the UV_254_ absorbance divided by the DOC concentration. On-site measurements of pH and conductivity were performed using a pH meter (Thermo Scientific Orion 4-star Benchtop pH meter, USA) and a Hach turbidimeter (2100 N model, USA), respectively. The samples were then transported to the laboratory as soon as possible to conduct the analytical procedures. A centrifuge (Z32 HK, Germany) was applied for centrifugation.

All of the THMs (TCM, BDCM, DBCM, and TBM) in methanol (2000 mg/L each) as the standard solutions were purchased from Sigma-Aldrich (Milwaukee, WI, USA). A GC system (Agilent 6850, USA) with a split/splitless injector and an electron capture detector (ECD) was used for the determination and gas chromatographic separation of the THMs. A 25 m × 0.32 mm capillary column coated with a 1.20 µm film of CP-Sil 13CB (86% methyl 14% phenyl siloxane) was used for compound separation. The initial oven temperature was set at 30 °C (held for 5 min) and then programmed at 10 °C min^−1^ to 140 °C (held for two minutes). The detector temperature was set at 300 °C. Ultrapure nitrogen (99.9999%, Air Products) was passed through a molecular sieve trap and an oxygen trap (CRS) at 30 mL/min and was used as a make-up gas for the ECD.

### Experimental procedure

Disinfection and herbal addition experiments were simulated in the laboratory. In all cases, reagent or higher-grade chemicals were used. The glassware used during this study was soaked in 10% nitric acid, rinsed with deionized water, and then dried. For the disinfection process, appropriate amounts of sodium hypochlorite (NaOCl) were added to the water samples and completely mixed for 30 minutes to obtain disinfectant levels of 0 (control), 0.5, 1.5 and 3 mg/L. Afterwards, appropriate amounts of herbal medicines in the form of a bath bag were added to the water samples. The bag was hung in the middle of the water sample to simulate the hot tub conditions in herbal spas. Different herbal bath bags of ginger (*Zingiber officinale*), Asian ginseng (*Panax ginseng*), mint (*Mentha spicata*), and rosemary (*Rosmarinus officinalis*) were used to simulate the herbal bath conditions. Fresh herbs were purchased from a local market and placed with a mass concentration of 2 or 10% in a cotton bag. Each bath bag was put into a beaker filled with laboratory chlorinated spa water. The beakers were covered by a watch glass to prevent contamination or loss of content. A magnetic stirring bar was placed in the beaker for 72 hours. During this experiment, neither the water nor herbs were exchanged. The same water was set up for comparison experiments without the bath bags. The temperature of the laboratory herbal spa water samples was kept constant at 39 °C ± 0.5, and the pH was adjusted to 7 ± 0.5 with the addition of 1 M HNO_3_ or NaOH and maintained until the end of the experiments. The water quality parameters and THM levels were regularly recorded during the experiments.

### THM extraction procedure

The THM extraction process was performed using a simple, environmentally friendly, rapid preconcentration and microextraction method, namely, dispersive liquid-liquid microextraction (DLLME) developed by Kozani *et al*.^77^.

A water sample (5.00 mL) was placed into a 10-mL conical-bottom glass centrifuge tube with a screw cap, and 1,2-dibromopropane was spiked as the internal standard. Acetone (0.50 mL, as the disperser solvent) containing 20.0 µL carbon disulfide (as the extraction solvent) was quickly injected into the solution using a 0.50-mL syringe (gas-tight; Hamilton, USA). After gentle shaking, a cloudy solution (a water–acetone–carbon disulfide mixture) was formed in the test tube. The mixture was then centrifuged for 1.0 minute at 6,000 rpm, causing the dispersed fine droplets of the extraction phase to settle to the bottom of the test tube. Approximately one µL of the settled phase was injected into the GC using a microsyringe.

### FTIR

An FTIR spectrometer (Perkin Elmer, Spectrum 100, USA) was employed to characterize the functional groups present in the various applied herbal medicines. FTIR can provide useful information for identifying the presence of major functional groups or chemical bonds in a molecular structure^78^ and has become increasingly popular for evaluating herbal qualities^79^. The plant material was thoroughly washed with tap and DI water to remove soil and other dirt and then completely dried using air. The dried plant material was converted into a powder using a heavy-duty blender. The powder was extracted with methanol according to the maceration method, and the extract was filtered using Whatman No. 1 filter paper. The filtrate was concentrated in a rotary evaporator at 40 °C. The concentrated extract was oven dried at 40 °C for three days and freeze-dried for 48 hours. For the FTIR analysis, a small quantity of the extract was dispersed in dry potassium bromide (KBr). The mixture was thoroughly mixed in a mortar and pressed at a pressure of six bars within two minutes to form a KBr thin disc. Then, the disc was placed in a sample cup with a diffuse reflectance accessory. The spectrum was obtained using an infrared spectrometer. The sample was scanned 16 times from 4000 to 400 cm^−1^ to increase the signal-to-noise ratio.

### Chlorine demand

The “chlorine demand” parameter is defined as the reduction in FRC with contact time due to its reaction with the various constituents in the water. The FRC level was measured at gradually increasing time intervals. The chlorine demand was calculated by subtracting the residual chlorine measured at the end of an experiment from the applied chlorine dose. The free chlorine was measured according to the Taiwan EPA method for spring water chemical analysis^80^ using a spectrophotometer (Genesys™ 10 S, WI, USA). Before beginning the laboratory experiments, all containers and glassware were cleaned with deionized water and heated at 450 °C for at least four hours to ensure that no chlorine demand was previously present.

### Data Availability

The datasets generated and analyzed during the current study are available from the corresponding author on reasonable request.

## References

[1] Hsu CS, Huang WZ, Wang HY (2011). Evaluation of disinfection efficiency between sodium hypochlorite and chlorine dioxide on spa water. Sustain. Environ. Res..

[2] Saslis-Lagoudakis CH (2012). Phylogenies reveal predictive power of traditional medicine in bioprospecting. Proc. Natl. Acad. Sci. USA.

[3] Hsu, C. S. & Huang, D. J. Disinfection of herbal spa pool using combined chlorine dioxide and sodium hypochlorite treatment. Environ. Monit. Assess. 187–34 (2015). 10.1007/s10661-014-4242-3 25632897

[4] HPA. In Management of Spa Pools: Controlling the Risk of Infection Available at http:// www.hpa.org.uk/publications/2006/spa_pools/ (London: Health Protection Agency, 2006).

[5] Zwiener C (2007). Drowning in disinfection byproducts? Assessing swimming pool water. Environ. Sci. Technol..

[6] Villanueva CM (2007). Bladder cancer and exposure to water disinfection by-products through ingestion, bathing, showering, and swimming in pools. Am. J. Epidemiol..

[7] Cantor KP (2010). Polymorphisms in GSTT1, GSTZ1, and CYP2E1, disinfection by-products, and risk of bladder cancer in Spain. Environ. Health Perspect..

[8] Chu H, Nieuwenhuijsen MJ (2002). Distribution and determinants of trihalomethane concentrations in indoor swimming pools. Occup. Environ. Med..

[9] Bull RJ (1995). Water chlorination: Essential process or cancer hazard. Fund. Appl. Toxicol..

[10] Weng SC, Blatchley III ER (2011). Disinfection by-product dynamics in a chlorinated, indoor swimming pool under conditions of heavy use: National swimming competition. Water Res..

[11] Righi E, Fantuzzi G, Predieri G, Aggazzotti G (2014). Bromate, chlorite, chlorate, haloacetic acids, and trihalomethanes occurrence in indoor swimming pool waters in Italy. Microchem. J..

[12] Yang CY, Chiu HF, Cheng MF, Tsai SS (1998). Chlorination of drinking water and cancer mortality in Taiwan. Environ. Res..

[13] Singer, P. C. *et al*. In Relative dominance of HAAs and THMs in treated drinking water. AWWA Research Foundation and American Water Works Association ISBN 1-58321-117-9 (2002).

[14] Daifullah AHM, Rizk MA, Aly HM, Yakout SM, Hassen MR (2011). Treatment of Some Organic Pollutants (THMs) using Activated Carbon Derived from Local agro-residues. Adv. Appl. Sci. Res..

[15] Morrison, R. D. & O’Sullivan, G. In Environmental forensics: Proceedings of the 2013 INEF conference, 247 pp (Royal Society of Chemistry, 2014).

[16] Pavelic P, Nicholson BC, Dillon PJ, Barry KE (2005). Fate of disinfection by-product in ground water during aquifer storage and recovery with reclaimed water. J. Contam. Hydrol..

[17] World Health Organization (WHO). Guidelines for Safe Recreational Water Environments, Swimming Pools, Spas and Similar Recreational Water Environments. **2**, pp. 146 (2006).

[18] Beech JA, Diaz R, Ordaz C, Palomeque B (1980). Nitrates, chlorates and trihalomethanes in swimming pool water. Am. J. Public Health.

[19] Sandel, B. B. Disinfection By-products in Swimming Pools and Spas (Technical Progress Report). Cheshire, Connecticut: Olin Corporation Research Center (1990).

[20] Fantuzzi, G., *et al* Occupational exposure to trihalomethanes in indoor swimming pools. C **264**(3), 257–265 (2001). 10.1016/s0048-9697(00)00722-1 11213196

[21] Aggazzotti G, Predieri G (1986). Survey of volatile halogenated organics (VHO) in Italy: Levels of VHO in drinking waters, surface waters and swimming pools. Water Res..

[22] Weaver WA (2009). Volatile disinfection by-product analysis from chlorinated indoor swimming pools. Water Res..

[23] Lindstrom AB, Pleil JD, Berkoff DC (1997). Alveolar breath sampling and analysis to assess trihalomethane exposures during competitive swimming training. Environ. Health Perspect..

[24] Chen MJ, Lin CH, Duh JM, Chou WS, Hsu HT (2011). Development of a multi-pathway probabilistic health risk assessment model for swimmers exposed to chloroform in indoor swimming pools. J. Hazard. Mater..

[25] Wang X, MI GL, Zhang X, Yang H, Xie Y (2014). Haloacetic acids in swimming pool and spa water in the United States and China. Front. Environ. Sci. Eng..

[26] Shukla Y, Singh M (2007). Cancer preventive properties of ginger: A brief review. Food Chem. Toxicol..

[27] Bode, A. M. & Dong, Z. The Amazing and Mighty Ginger. In: Benzie IFF, Wachtel-Galor S, editors. Herbal Medicine: Biomolecular and Clinical Aspects. 2nd edition. Boca Raton (FL): CRC Press/Taylor & Francis; Chapter 7. Available from: https://www.ncbi.nlm.nih.gov/books/NBK92775/ (2011).

[28] Shan Y, Xh X, Up J (1994). Effects of ginsenosides on myocardial ischemia/reperfusion damage in open heart surgery patients. Chung Hua I Hsueh Tsa Chih (Med J China).

[29] Rimar S, Lee-Mengel M, Gillis CN (1996). Pulmonary protective and vasodilator effects of a standardized Panax ginseng preparation following artificial gastric digestion. Pulm. Pharmacol..

[30] Park KJ, Vohnikova Z, Brod FPR (2002). Evaluation of drying parameters and desorption isotherms of garden mint leaves (Mentha crispa L.). J. Food Eng..

[31] AI-Sereiti MR, Abu-Amer KM, Sen P (1999). Pharmacology of rosemary (Rosmarinus oificinalis Linn.) and its therapeutic potentials. Indian J. Exp. Biol..

[32] Dahmani B, Chab M (2011). Study of Membrane Fouling and Trihalomethane Formation in Reverse Osmosis Desalination Pilot Unit. J Membra. Sci. Technol..

[33] US EPA. National primary drinking water regulations: Stage 2 disinfectants and disinfection byproducts rule (stage 2 DBPR), final rule, Federal register **71**, 387–493 (2006).

[34] USEPA. Risk Assessment Guidance for Superfund. Vol.1 Human Health Evaluation Manual. EPA/540/1-89/002. (1989).

[35] Aprea MC (2010). Disinfection of swimming pools with chlorine and derivatives: formation of organochlorinated and organobrominated compounds and exposure of pool personnel and swimmers. Natural Sci..

[36] Susan D (2010). What’s in the Pool? A Comprehensive Identification of Disinfection By-products and Assessment of Mutagenicity of Chlorinated and Brominated Swimming Pool Water. Environ. Health Perspect..

[37] Soontornchai, S., Panyakapo, M. & Paopuree, P. Life time cancer risk assessment from exposure of trihalomethanes in tree swimming pool types. Proceedings of the 11th international conference on Environmental Science and Technology, Chania, Crete, Greece (2009).

[38] Gómez-López VM, Lannoo AS, Gil MI, Allende A (2014). Minimum free chlorine residual level required for the inactivation of Escherichia coli O157:H7 and trihalomethane generation during dynamic washing of fresh-cut spinach. Food Control.

[39] ÖzdemJr K (2014). Characterization of Natural Organic Matter in Conventional Water Treatment Processes and Evaluation of THM Formation with Chlorine. Scientific. World J..

[40] E. P. A. Drinking Water Guidance on Disinfection By-Products. Advice Note No. 4, version 2, Disinfection By-Products in Drinking Water, Office of Environmental Enforcement: available at http://www.epa.ie/pubs/advice/drinkingwater/DrinkingWaterGuide4_v8. pdf (2012).

[41] Tseng WV, Lou JC, Han JY (2013). A Study of Removing SUVA and Trihalomethanes by Biological Activated Carbon. Int. J. Environ. Chem. Ecol. Geo. Geophys. Eng..

[42] Korshin GV, Li CW, Benjamin MM (1997). The decrease of UV absorbance as an indicator of TOX formation. Water Res..

[43] Lee JW, Do JH, Lee SK, Yang JW (2000). Determination of total phenolic compounds from Korean red ginseng, and their extraction conditions. J. Ginseng Res..

[44] Chung IM, Kim JW, Seguin P, Jun YM, Kim SH (2012). Ginsenosides and phenolics in fresh and processed Korean ginseng (Panax ginseng C. A. Meyer): Effects of cultivation location, year, and storage period. Food Chem..

[45] Kong YH, Lee YC, Choi SY (2009). Neuroprotective and anti-inflammatory effects of phenolic compounds in Panax ginseng C.A. Meyer. J Ginseng Res..

[46] Chung IM, Lim JJ, Ahn MS, Jeong HN, An TJ (2016). Comparative phenolic compound profiles and antioxidative activity of the fruit, leaves, and roots of Korean ginseng (Panax ginseng Meyer) according to cultivation years. J. Ginseng Res..

[47] Fabris R, Chowa CWK, Drikas M, Eikebrokk B (2008). Comparison of NOM character in selected Australian and Norwegian drinking waters. Water Res..

[48] Gallard H, von Gunten U (2002). Chlorination of natural organic matter: Kinetics of chlorination and of THM formation. Water Res..

[49] Cui S, Wu J, Wang J, Wang X (2017). Discrimination of American ginseng and Asian ginseng using electronic nose and gas chromatography mass spectrometry coupled with chemometrics. J. Ginseng Res..

[50] Kwon JH (1990). Chemical Constituents of Panax ginseng Exposed to y Irradiation. J. Agric. Food Chem..

[51] Chang, Y. H. Effects of Extrusion and Baking Processes on Ginsenosides in Wheat Flour-ginseng Powder Blends. Michigan State University, ProQuest pp.176 (2008).

[52] Lin W (2016). Oxidation of aniline aerofloat in flotation wastewater by sodium hypochlorite solution. Environ. Sci. Pollut. Res..

[53] Gagnon GA (1997). Carboxylic acids: formation and removal in full-scale plants. J. Am. Water Works Assn..

[54] Sarathy SR, Mohseni M (2007). The impact of UV/H2O2 advanced oxidation on molecular size distribution of chromophoric natural organic matter. Environ. Sci. Technol..

[55] Lamsal R, Walsh ME, Gagnon GA (2011). Comparison of advanced oxidation processes for the removal of natural organic matter. Water Res..

[56] Tomoda M, Shimada K, Konno C, Sugiyama K, Hikino H (1984). Partial Structure of Panaxan A, A Hypoglycaemic Glycan of Panax ginseng Roots. Planta Med..

[57] Lin S, Evans R (1974). Chlorine demand study of secondary sewage effluents. Water Sewage Works.

[58] Helbling DE, VanBriesen JM (2007). Free chlorine demand and cell survival of microbial suspensions. Water Res..

[59] Kanan A, Karanfil T (2011). Formation of disinfection by-products in indoor swimming pool water: The contribution from filling water natural organic matter and swimmer body fluids. Water Res..

[60] Rodríguez-Murillo JC, Zobrist J, Filella M (2015). Temporal trends in organic carbon content in the main Swiss rivers, 1974–2010. Sci. Total Environ..

[61] Krasner SW, McGuire MJ, Jacangelo NL, Patania KM, Aieta EM (1989). The occurrence of disinfection by-products in US drinking water. J. Am. Water Works Assoc..

[62] Qi Y, Shang C, Lo IMC (2004). Formation of haloacetic acids during monochloramination. Water Res..

[63] Huang WY, Cai YZ, Zhang Y (2009). Natural Phenolic Compounds From Medicinal Herbs and Dietary Plants: Potential Use for Cancer Prevention. ‎Nutr. Cancer.

[64] Dyck R, Sadiq R, Rodriguez MJ, Simard S, Tardif R (2011). Trihalomethane exposures in indoor swimming pools: a level III fugacity model. Water Res..

[65] Montesinos I, Gallego M (2014). How the Inclusion of Treated Water in Beverages Influences the Appearance of Halogenated Volatile Organic Compounds. J. Agric. Food Chem..

[66] Dos Santos MS, Martendal E, Carasek E (2011). Determination of THMs in soft drink by solid-phase microextraction and gas chromatography. Food Chem..

[67] Yuita K (1983). Iodine, bromine and chlorine contents in soils and plants of Japan. Soil Sci. Plant Nutr..

[68] Yuita A, Nobusawa Y, Shibuya M, Aso S (1982). Iodine, bromine and chlorine contents in soils and plants of Japan. I. Iodine, bromine and chlorine contents in soils and plants of the basin of the Miomote River. Soil Sci. Plant Nutr..

[69] Wang GS, Deng YC, Lin TF (2007). Cancer risk assessment from trihalomethanes in drinking water. Sci. Total Environ..

[70] Huang W, Chang C, Shih F (2009). Disinfection by-product formation and mutagenic assay caused by preozonation of groundwater containing bromide. Environ. Monit. Assess..

[71] Jain PK, Soni A, Jain P, Bhawsar. J (2016). Phytochemical analysis of Mentha spicata plant extract using UV-VIS, FTIR and GC/MS technique. J. Chem. Pharm. Res..

[72] Stevenson, F. J. H. C. Genesis, Composition, Reactions. 2nd ed.,Wiley, New York (1994).

[73] Silverstein, R.M. & Webster, F. X. Spectrometric Identification of Organic Compounds. Wiley, New York (1998).

[74] Zhang X (2015). Concentration levels of disinfection by-products in 14 swimming pools of China. Front. Environ. Sci. Eng..

[75] Nelemans PJ (1994). Swimming and the risk of cutaneous melanoma. Melanoma Res..

[76] Taiwan EPA. Class I Public Water Quality Standards. Taiwan Environmental Protection Administration, Available at http://www.watertec.com/epa/lls.htm (2011).

[77] Kozani R, Assadi V, Shemirani F, Milani Hosseini MR, Jamali MR (2007). Determination of trihalomethanes in drinking water by dispersive liquid–liquid microextraction then gas chromatography with electron-capture detection. Chromatographia.

[78] Liu T (2011). Application of ionic liquids based microwave-assisted simultaneous extraction of carnosic acid, rosmarinic acid and essential oil from Rosmarinus officinalis. J. Chromatogr. A.

[79] Joshi, D. D. Herbal Drugs and Fingerprints: Evidence Based Herbal Drugs. Springer India, 10.1007/978-81-322-0804-4_7 (2012).

[80] TEPA, C I Public Water Quality Standards. Taiwan Environmental Protection Administration, Taipei, Taiwan. Available at http://www.watertec.com/epa/lls.htm (2009).

